# UCC118 supplementation reduces exercise‐induced gastrointestinal permeability and remodels the gut microbiome in healthy humans

**DOI:** 10.14814/phy2.14276

**Published:** 2019-11-23

**Authors:** Christopher L. Axelrod, Connery J. Brennan, Gail Cresci, Deborah Paul, Michaela Hull, Ciarán E. Fealy, John P. Kirwan

**Affiliations:** ^1^ Department of Inflammation and Immunity Lerner Research Institute Cleveland Clinic Foundation Cleveland Ohio; ^2^ Integrated Physiology and Molecular Medicine Laboratory Pennington Biomedical Research Center Baton Rouge Los Angeles; ^3^ Department of Translational Services Pennington Biomedical Research Center Baton Rouge Los Angeles

**Keywords:** Exercise, gastrointestinal permeability, microbiome, probiotic supplementation, UCC118, *Verrucomicrobia*

## Abstract

Dysregulation of gut microbiota and intestinal barrier function has emerged as potential mechanisms underlying digestive diseases, yet targeted therapies are lacking The purpose of this investigation was to assess the efficacy of UCC118, a characterized probiotic strain, on exercise‐induced GI permeability in healthy humans. In a randomized, double‐blind, placebo‐controlled crossover study, seven healthy adults received 4 weeks of daily UCC118 or placebo supplementation. GI hyperpermeability was induced by strenuous treadmill running performed before and after each supplementation period. While running, participants ingested 5 g of lactulose, rhamnose, and sucrose. Urine was collected before, immediately after, and every hour for 5 h after exercise to assess GI permeability. Metagenomic sequencing was performed on fecal homogenates collected prior to exercise to identify changes in microbial diversity and taxon abundances. Inflammatory biomarkers were assessed from blood and fecal homogenates collected prior to and immediately following the cessation of exercise. Exercise significantly induced intestinal permeability of lactulose, rhamnose, and sucrose (*P* < 0.001). UCC118 significantly reduced sucrose (Δ = −0.38 ± 0.13 vs. 1.69 ± 0.79; *P* < 0.05) recovery, with no substantial change in lactulose (Δ = −0.07 ± 0.23 vs. 0.35 ± 0.15; *P* = 0.16) or rhamnose (Δ = −0.06 ± 0.22 vs. 0.48 ± 0.28; *P* = 0.22). Taxonomic sequencing revealed 99 differentially regulated bacteria spanning 6 taxonomic ranks (*P* < 0.05) after UCC118 supplementation. No differences in plasma IL‐6 or fecal zonulin were observed after UCC118 supplementation. The results described herein provide proof of principle that 4 weeks of UCC118 supplementation attenuated exercise‐induced intestinal hyperpermeability. Further research is warranted to investigate the as‐yet‐to‐be defined molecular processes of intestinal hyperpermeability and the effects of probiotic supplementation.

## Abstract 2

Dysregulation of gut microbiota and intestinal barrier function have emerged as potential mechanisms underlying digestive diseases, yet targeted therapies are lacking. In a randomized, double‐blind, placebo‐controlled crossover study, 7 healthy adults 30 received 4 weeks of daily UCC118 or placebo supplementation. UCC118 significantly reduced sucrose recovery. Taxonomic sequencing revealed 99 differentially regulated gut microbes by UCC118. The results herein provide proof of principle that UCC118 supplementation can reduce intestinal hyperpermeability.

## Graphical abstract

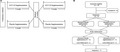

## Introduction

Approximately 60 to 70 million individuals in the United States suffer from digestive disorders, including Crohn’s disease, Celiac disease, and irritable bowel syndrome (IBS) (National Institutes of Health 2009). Clinical signs are variable, but often present as persistent diarrhea, abdominal cramping and pain, excessive bloating and flatulence, nausea and vomiting, or rectal bleeding. Interestingly, the gastrointestinal symptoms observed in IBS are similar to those observed in endurance athletes, where splanchnic hypoperfusion is believed to comprise intestinal barrier function. Though the underlying mechanisms of these complications in both patients with GI disease and endurance athletes remain unclear, perturbations in the GI tract are frequently documented (Arrieta et al. 2006), and include a breakdown in the intestinal epithelial barrier leading to increased solute permeability (Bischoff et al. 2014).

More recently, remodeling of intestinal microbiota has been implicated in the pathophysiology of digestive diseases (Gorkiewicz and Moschen 2018). As biologics are limited and/or have harsh side effects, there is increased interest in modifying the gut microbiome with probiotics as a means to manage these diseases. While existing evidence demonstrates efficacy of some probiotics in the management of certain disorders, the benefits appear to be strain specific and not transferable to generalized GI complaints (Lamprecht et al. 2012; Whelan and Quigley 2013; Ghouri et al. 2014). UCC118^®^ is a strain of *Lactobacillus salivarius* (*Lb. salivarius*) isolated from the human terminal ileum with characteristics desirable for the manufacturing of an orally administered probiotic species (Dunne et al. 2001). These include enhanced adhesive properties in human intestinal epithelial cells (IECs) (Neville and O'Toole 2010), direct microbial inhibition, bile resistance, acid tolerance, and inflammatory inhibition (Claesson et al. 2006; O'Hara et al. 2006). Furthermore, UCC118 has been used to improve glycemic control and pregnancy outcome among women with gestational diabetes. Supplementation was safe, tolerable, and led to an attenuation of maternal lipid responses (Lindsay et al. 2015). Preliminary in vitro and in vivo evidence suggest that UCC118 is a safe, well‐tolerated probiotic strain with therapeutic potential for the management of GI symptoms and disorders. Therefore, the purpose of this investigation was to determine the effect of a *Lb. salivarius* strain, UCC118, on GI permeability in healthy humans.

## Materials and Methods

### Subjects

**Figure 1:**
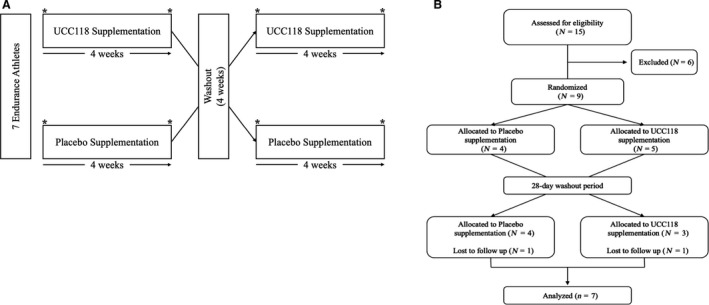
(A) Overview of study design. Following randomization, participants received 4 weeks of daily probiotic or placebo supplementation. Drug administration was followed by a 4 week washout period prior to the crossover assessment period. GI permeability, body composition, blood chemistry, and fecal microbiota were assessed before and after each 4‐week intervention period. (B) CONSORT flow diagram. Nine participants were randomized to placebo (*N* = 4) or UCC118 (*N* = 5). Two participants, one from each treatment arm, were lost to follow‐up during the 28‐day washout period. All seven of the completed subjects were included in the analysis of primary and secondary study outcomes.

Seven trained endurance athletes aged 18–45 years completed a randomized, double‐blind, placebo‐controlled crossover study (Fig. 1A). Prior to randomization, study eligibility was assessed, which included a medical screening for heart, kidney, liver, thyroid, intestinal, and pulmonary diseases and medications known to affect the primary outcome variables. Inclusionary criterion was as follows: maximal aerobic capacity (VO_2MAX_) ≥50 mL/kg/min, no current use of oral contraceptives, nonsmoking, and no dietary or probiotic supplementation within 1 month of entry into the study. Participants were weight stable for at least six months prior to enrollment. The study was approved by the Cleveland Clinic Institutional Review Board (IRB# 15‐1311). All subjects provided written informed consent in accordance with the ethical standards of the responsible institutional or regional committee on human experimentation or in accordance with the Helsinki Declaration of 1975 as revised in 1983. Descriptive characteristics of study participants are displayed in Table 1.

**Table 1:** Participant characteristics.

	Mean	SD
Age (year)	31.0	2.3
Weight (kg)	71.2	14.1
BMI (kg/m^2^)	24.3	3.4
Body fat (%)	20.1	5.9
HR_MAX_ (bpm)	181.1	7.9
VO_2MAX_ (mL·kg^−1^·min^−1^)	57.9	4.5
Systolic blood pressure	124.6	12.1
Diastolic blood pressure	72.4	4.7
Glucose (mg/dL)	87.6	7.2
Triglyceride (mg/dL)	63.2	14.4
Cholesterol (mg/dL)	178.2	29.3
HDL cholesterol (mg/dL)	75.7	18.4
LDL cholesterol (mg/dL)	90.0	27.7
WBC (k/*µ*L)	4.6	0.5
RBC (m/*µ*L)	4.9	0.3
Hemoglobin (g/dL)	15.3	1.0
Hematocrit (%)	44.4	1.4

### Procedures

Following randomization, participants underwent a 2‐week weight and diet stabilization period without treatment. Participants reported to the Clinical Research Unit (CRU) at 0600 after a 10‐h fast and 24‐h abstention from alcohol, caffeine, and structured exercise. Participants then provided a fasting blood and urine sample. Additionally, participants collected stool samples for 48 h prior to testing and provided the samples upon arrival to the CRU. Participants were instructed to use standardized fecal collection kits and to place them into a refrigerated cooler until arrival. One hour prior to exercise, participants received a standardized meal (5.97 ± 0.5 kcal/kg body mass) consisting of 50% carbohydrate (52.1 g), 15% protein (15.6 g), and 35% fat (16.2 g). Participants then initiated 2 h of continuous aerobic exercise at 60% of VO_2MAX_ (~75% of maximal heart rate) to induce hyperpermeability of the GI tract as described previously (Oktedalen et al. 1992; Pals et al. 1997; Lambert et al. 2001; Zuhl et al. 2014). Twenty minutes after the onset of exercise, participants straddled the sides of the treadmill, and were provided a 100 mL solution containing 5 g lactulose (Kristalose^®^; Cumberland Pharmaceuticals), 5 g rhamnose (L‐Rhamnose; Sigma‐Aldrich) and 5 g sucrose (Sucrose; Sigma‐Aldrich). Participants consumed water ad libitum during the 2‐h exercise period. Immediately upon cessation of exercise, additional blood and urine samples were obtained. Urine was collected every hour over a 5‐h period. Temperature (24.9 ± 1.3°C) and relative humidity (30.8 ± 1.8%) were measured and adjusted during the 2‐h continuous exercise session.

### Supplementation

Prior to discharge, subjects received a 4‐week supply of 200 mg capsules containing the *Lactobacillus salivarius* UCC118 (2 × 10^8^ CFU/capsule) or placebo (corn starch with magnesium stearate). Participants were instructed to consume one capsule daily with fluid, and to store the probiotics at 4°C for the duration of study. Participants then returned 4 weeks after the initiation of supplementation for posttesting, and all procedures were repeated. Drug compliance was measured by collection and counting of capsule containers. Between testing phases, there was a 4‐week supplementation washout period. Participants were instructed to continue normal lifestyle patterns during this time while refraining from dietary and probiotic supplementation. UCC118 and placebo capsules were manufactured and provided by Metagenics, Inc. (Aliso Viejo, CA).

### Aerobic capacity

VO_2MAX_ was determined using a modified Bruce protocol as described previously (Axelrod et al. 2019). Briefly, exhaled air was continuously sampled online with the use of an automated system (Jaeger Oxycon Pro; Viasys, Yorba Linda, CA). The test was deemed to be maximal if at least three of the following criteria were satisfied: a respiratory exchange ratio of ≥1.10, a leveling off in oxygen consumption with increasing workloads, volitional fatigue, or a heart rate greater than or equal to age‐predicted maximum.

### Body composition

Body composition was determined after a 10‐h overnight fast using whole body air displacement plethysmography (BOD POD^®^, COSMED USA Inc.; Concord, CA). The BOD POD and weight scale were calibrated prior to each individual test. All subjects wore minimal, form fitting clothing and a swim cap during testing according to device guidelines. During measurement, subjects were instructed to sit still with their hands resting upon their thighs and breathe normally. Whole body density was used to determine percent body fat using the Siri equation (Siri 1993).

### Gastrointestinal permeability

Upon collection, total urinary output (mL) was measured and recorded. Twenty milliliter urine aliquots from each time point were then stored at −20°C until analysis. For analysis, urine samples were thawed and stirred for 1 min using a vortex mixer. Samples were then centrifuged at 4000*g* for 4 min to remove sediment. After centrifugation, samples were diluted 1:5 in acetonitrile and quantified using High Performance Liquid Chromatography On‐line Electrospray Ionization Tandem Mass Spectrometry (HPLC/ESI/MS/MS). Raffinose was used as the internal standard and added to each of the samples at a final concentration of 10 µg/mL. Briefly, a C18 column (2 × 150 mm, Prodigy, 5 *µ*m, Phenomenex) was used for separation on a Shimadzu HPLC. Mobile phases were water containing 0.2% acetic acid and acetonitrile containing 0.2% acetic acid. The HPLC eluent was directly injected into a triple quadrupole mass spectrometer (Shimadzu LCMS‐8050) and the analytes were ionized. Sugars were quantified using Selected Reaction Monitoring (SRM; *m*/*z*) as follows: 341/161 (lactulose), 163/103 (sucrose), and 341/179 (rhamnose). *Labsolutions* software (Shimadzu, Kyoto, Japan) was used to process the data, and the internal standard calibration curves were used to calculate the concentrations of the three sugar alcohols in urine samples. The final concentrations determined by HPLC/ESI/MS/MS were then normalized by amount of sugar ingested (g), body weight (kg), and urinary output (mL), to quantify GI permeability.

### Core temperature

Immediately upon arrival at the CRU, participants ingested a plastic, encapsulated body temperature probe (HQ Inc.; Palmetto, FL). Data were transmitted to a wireless monitoring device in real time via magnetic flux and recorded at baseline, and every 10 min throughout the exercise session.

### Fecal collection

Feces were collected 48 h prior to testing, starting with the first morning passage. When participants passed a stool, they collected it in a preweighed plastic container with a prelabeled stomacher bag as a liner. Upon arrival to the CRU, stool homogenates were derived by combining all stool samples into a single stomacher bag, and mixed for 1 min at high speed. If stool was too hard to mix, a measured amount of sterile water was added and remixed to ensure adequate homogenization. Aliquots for fecal microbiota analysis and zonulin were then obtained and stored at −80°C until assay.

### Fecal zonulin

Fecal zonulin concentrations were determined using a commercially available competitive binding enzyme‐linked immunosorbent assay (ELISA) with the following modifications (ALPCO^®^; Salem, NH). Fifteen milligrams of stool homogenate was diluted 1:50 in extraction buffer using a sample applicator system (IDK; Bensheim, DE). The resulting homogenates were vortexed for 10 min, and placed on ice to allow any remaining debris to settle. About 1 and 5 ng/mL recombinant zonulin protein were used as an internal standard. The intraassay coefficient of variation was 3.3%.

### Fecal DNA extraction

Approximately 150 *µ*L of fecal homogenate was extracted for next‐generation shotgun metagenomic sequencing of microbial taxon (Illumina). Briefly, DNA was extracted from fecal samples using the Machery‐Nagel NucleoSpin^®^ Soil kit. Each 250–500 *µ*L fecal sample was aliquoted into NucleoSpin^®^ Bead Tube Type A, containing ceramic beads. Seven hundred microliters of lysis buffer and 150 *µ*L of SX Enhancer (to ensure largest possible DNA yield) were added to each sample before vortexing at room temperature for 5 min. Samples were centrifuged for 2 min at 11,000*g*. A third lysis buffer was added to samples before vortexing for 5 sec, and then incubated at 4°C for 5 min. Following 1 min centrifugation at 11,000*g*, supernatant was transferred into new collection tubes and combined with 250 *µ*L of Buffer SB. Soil columns were placed into collection tubes, and 550 *µ*L of sample was loaded into each column. Samples were centrifuged for 1 min at 11,000*g*. Flow through tubes were discarded and columns were placed into new collection tubes. Remaining sample was added onto the columns. Samples were washed four times per manufacturer’s recommendations. Following the fourth wash, columns were placed into collection tubes and centrifuged for dry run for 2 min at 11,000*g*. DNA was then eluted per manufacturer’s recommendations, including the addition of Buffer SE, depending on yield. After incubating at room temperature, samples were centrifuged for 30 sec at 11,000*g* and prepped for quality verification performed on 1% Agarose Gel, and DNA quantification using the Qubit dsDNA HS Assay Kit.

### Fecal microbiota sequencing

Next‐generation metagenomic sequencing was performed on purified fecal DNA extracts using the Illumina HiSeq 4000 (Illumina, Inc.; San Diego, CA) platform with an average read of 120–130 mol/L paired ends per sample (60–65 mol/L per direction). ZymoBIOMICS™ (Zymo Research; Irvine, CA) and sterile dH_2_O were used as positive and negative controls, respectively. Raw read counts were obtained for each of the identified taxa within the sample. Low complexity reads were removed to reduce background before assigning the taxonomic groups. A custom reference genome collection was generated from the larger NCBI nucleotide database to compile all identifiable taxa. Any taxa with an abundance of <5 reads were filtered. Approximately 4100 taxa (abundance >5 reads) were identified within the fecal samples. Reads passing the initial quality thresholds were then clustered against the reference sequence collection described above, identifying and assigning taxa. Taxa were profiled by aligning the reads against the reference sequence collection. Ambiguously mapped reads were reassigned to ensure all reads were mapped appropriately. The abundance for each marker representing a specific taxa was estimated as reads per kilo (RPK) base units, and then normalized by the total number of reads in the metagenome. Indices of bacterial diversity were calculated as described previously (Kim et al. 2017).

### Blood analyses

Blood samples were obtained from the antecubital vein at screening, as well as both before and immediately after the cessation of each exercise session. Samples were collected in 10 mL BD Vacutainer^®^ tubes treated with K_2_ EDTA. Whole blood was then centrifuged for 10 min at 3000*g* at 4°C. At screening, plasma samples were immediately assayed clinically for blood metabolites, lipids, and complete cell counts. During testing visits, isolated plasma was drawn off in 500 *μ*L aliquots and stored at −80°C until the time of analysis. Plasma concentrations of interleukin 6 (IL‐6) were then assayed using a commercially available ELISA (R&D Systems^®^, Minneapolis, MN) as per the manufacturers guidelines. The sensitivity of the assay was 0.7 pg/mL. The intraassay coefficient of variation was 3.5%.

### Statistical analysis

The primary outcome of this study was change in GI permeability following 4 weeks of supplementation with placebo or UCC118. A priori power analysis based on a previous investigation from healthy runners (Karhu et al. 2017) estimated approximately 6 would be needed to obtain statistical power at the recommended .80 level based upon the mean, between‐groups comparison effect size (*d* = 1.2). A two‐way, repeated measures analysis of variance (RM ANOVA) was used to assess differences in GI permeability. Incremental area under the curve sugar excretion was calculated using the trapezoidal method. Main effects of time and time x group were assessed post hoc using Tukey’s range test. Shapiro–Wilk’s test was used to assess normality of distribution. *F* test was used to compare sample variances. Welch’s *t*‐test was used for statistical assessments containing unequal variances. The secondary outcome of this study was changes in fecal microbiota and markers of inflammation following 4 weeks of probiotic supplementation. Changes in fecal microbiota abundance pre to postintervention were assessed using the Wald test. The false discovery rate (FDR) adjusted *P*‐values were derived using the Benjamini–Hochberg procedure. Between‐group differences in protein concentrations were assessed using a paired samples *t*‐test. The level of significance was set at *P* < 0.05.

## Results

**Figure 2:**
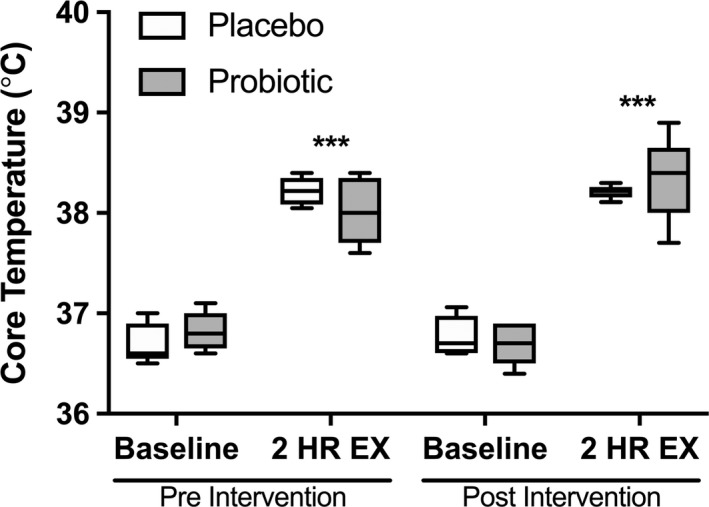
The effect of probiotic supplementation on core temperature regulation. Core temperature (°C) was measured from the digestive tract at rest and during the final 10 min of exercise. *(*P* < 0.05) indicates a significant time effect. ^#^(*P* < 0.05) indicates a significant treatment effect. Data are presented as the mean ± SE.

**Figure 3:**
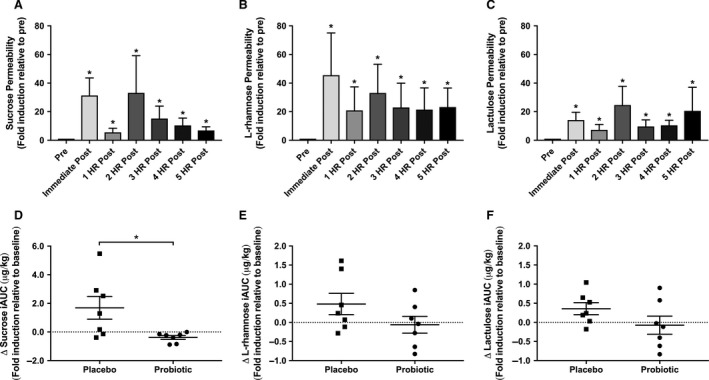
The effect of probiotic supplementation on GI permeability. Urinary excretion (*µ*g CHO/kg BW) of (A) sucrose, (B) L‐rhamnose, and (C) lactulose was determined prior to and immediately after exercise, and every 60 min over a 5 h recovery period. *(*P* < 0.05) indicates significant time effect. Change in (D) sucrose, (E) L‐rhamnose, and (F) lactulose permeability expressed as an incremental area under the curve (iAUC) after supplementation with placebo or UCC118. ^#^(*P* < 0.05) indicates a significant treatment effect. Data are presented as the mean ± standard error.

Seven participants completed the study protocol and were analyzed for primary and secondary outcomes (Fig. 1B). Exercise significantly increased core body temperature (Fig. 2; *P* < 0.001), with no differences observed between groups. Exercise also significantly induced GI hyperpermeability of sucrose (ES = 2.4; *P* < 0.001) (Fig. 3A), lactulose (ES = 1.1; *P* < 0.001) (Fig. 3B), and rhamnose (ES = 1.5; *P* < 0.001) (Fig. 3C; all *P* < 0.001). This effect was sustained across all recovery time points for all sugars (all *P* < 0.001). Supplementation with UCC118 significantly reduced net hyperpermeability of sucrose (Fig. 3D; Δ UCC118 = −38% vs. Δ placebo = 169%, ES = 1.38; *P* = 0.029), while not altering lactulose (Fig. 3E) or rhamnose (Fig. 3F) excretion (*P* = 0.149 and 0.216, respectively). Additionally, the lactulose/rhamnose recovery ratio was assessed (data not shown), which was unaffected by UCC118 supplementation (*P* = 0.15).

**Figure 4:**
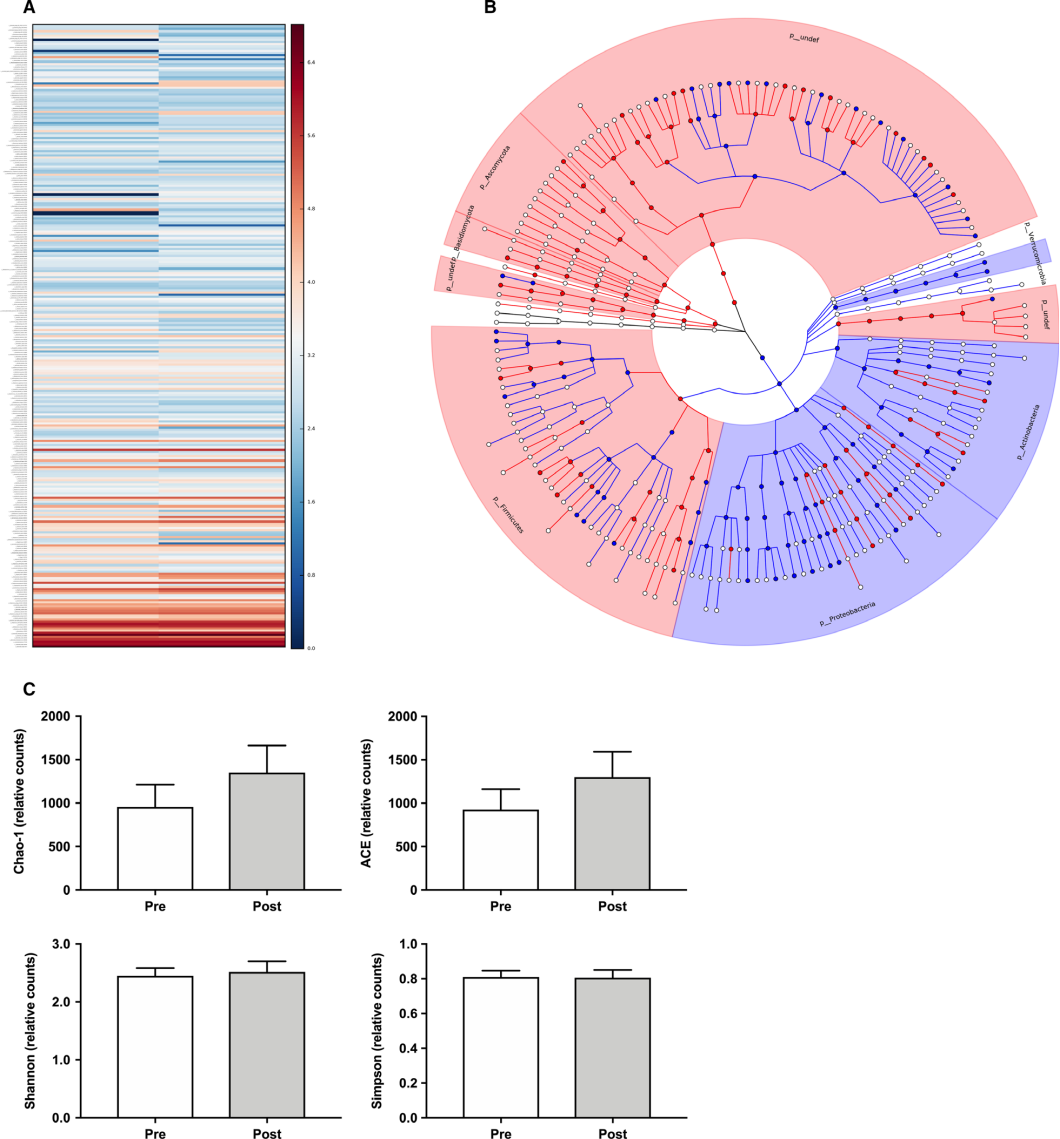
The effect of UCC118 on intestinal microbiota and bacterial diversity. (A) Heat map, and (B) circular ideogram visualizing differentially regulated fecal microbiota before and after treatment with UCC118. (C) Indices of microbial diversity and species richness before and after treatment with UCC118. *(*P* < 0.05) indicates significant treatment effect. Data are presented as the mean ± standard error.

Shotgun metagenomic sequencing of fecal homogenates revealed 99 differentially regulated microorganisms (*q* < 0.05) spanning six taxonomic classifications (Table S1; https://doi.org/10.6084/m9.figshare.8316512.v1). A heat map (Fig. 4A) and circular ideogram were then constructed to visualize changes and identify relationships spanning multiple taxonomic ranks (Fig. 4B). A significant reduction in the phylum *Verrucomicrobia* (*q* < 0.001) was observed, which carried through the class (*Verrucomicrobiae*), order (Verrucomicrobiales), family (*Verrucomicrobiaceae*), genus (*Prosthecobacter*), and species (*fusiformis*) taxon (Table 2). Furthermore, the effects of UCC118 on indices of bacterial diversity were characterized (Fig. 4C). No significant changes in Chao‐1 (*P* = 0.47), ACE (*P* = 0.46), Shannon (*P* = 0.74), and Simpson (*P* = 0.94) were observed.

**Table 2:** Effect of probiotic supplementation on phylum *Verrucomicrobia*.

Taxonomic classification	Name	Baseline (% abundance)	UCC118 (% abundance)	Fold change (Log2)	*q*‐value
Kingdom	Bacteria	84.552	85.131	0.050	0.909
Phylum	Verrucomicrobia	0.148	0.004	−5.390	0.001
Class	Verrucomicrobiae	0.167	0.003	−5.724	0.002
Order	Verrucomicrobiales	0.145	0.003	−5.557	0.004
Family	Verrucomicrobiaceae	0.169	0.003	−5.973	0.001
Genus	Prosthecobacter	0.144	0.003	−5.590	0.004
Species	fusiformis	0.051	0.000	−6.617	0.006

**Figure 5:**
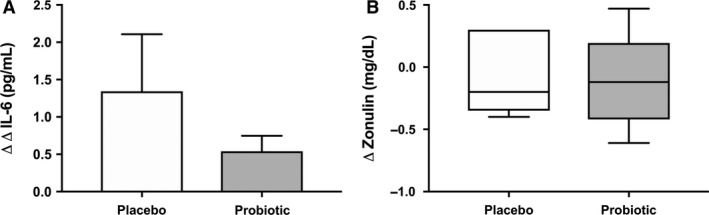
Changes in (A) circulating IL‐6 (ΔΔ; pre to postexercise, pre to postintervention) and (B) fecal zonulin (Δ; pre to postintervention) between treatment groups. *(*P* < 0.05) indicates significant treatment effect. Data are presented as the mean ± standard error.

Finally, probiotic supplementation did not alter basal or exercise‐induced IL‐6 (Fig. 5A) (*P* = 0.40). Fecal zonulin also remained unchanged after treatment (Fig. 4B) (*P* = 0.85).

## Discussion

In this study, we provide proof of principle that prophylactic supplementation with UCC118 reduced exercise‐induced intestinal hyperpermeability in healthy adults. Supplementation with UCC118 also remodeled the gut microbiome, without inducing notable changes in markers of inflammation or tight junction regulation. These data suggest orchestrating changes in the composition of gut microbiota is one way that UCC118 exerts its protective effect against GI hyperpermeability.

Increased intestinal permeability and gut dysbiosis are associated with digestive diseases, as exemplified in Celiac disease (Duerksen et al. 2005) and Crohn’s disease (Munkholm et al. 1994), as well as IBS (Zhou et al. 2009). Probiotic supplementation has emerged as a promising treatment target, though few bacterial strains have demonstrated efficacy in randomized controlled trials (Kajander et al. 2008; West et al. 2014). While some have observed marked improvements in GI permeability and function (Rosenfeldt et al. 2004; Lamprecht et al. 2012), others have demonstrated weaker to limited benefit (Leber et al. 2012; Shing et al. 2014). Overall integrity of design, length of supplementation, probiotic strain efficacy, and population differences limit comparison to the results obtained herein. Nonmetabolized disaccharide probes, such as lactulose and L‐rhamnose, are widely used as a clinical diagnostic for the assessment of intestinal permeability (van Elburg et al., 1995). Under nonpathologic conditions, less than 3% of lactulose is absorbed, and is primarily digested in the colon (Clausen and Mortensen 1997). Thus, hyperexcretion of lactulose largely represents damage to the distal intestinal tract (Mishra and Makharia 2012). Sucrose is rapidly metabolized in the small intestine after passage from the stomach, which allows for its use as a marker of upper GI or gastroduodenal permeability (Kawabata et al. 1998). Herein, sucrose permeability, rather than lactulose, L‐rhamnose, or the ratio of lactulose to L‐rhamnose, was reduced after UCC118 supplementation. Thus, these data suggest that UCC118 may function by reducing or protecting against exercise‐induced small intestinal permeability.

Multiple strains of *Lactobacilli* have been tested in vitro and in vivo for the prevention and treatment of GI disturbances (Johansson et al. 1993; Bernet et al. 1994; Rousseaux et al. 2007; Lievin‐Le Moal and Servin 2014). Network analysis of the changes in microbial abundances led to the identification of phylum *Verrucomicrobia*, which was markedly reduced and observed in all lower taxonomic ranks after UCC118 supplementation. The phyla *Verrucomicrobia* is lowly expressed throughout the human GI tract, with exception to the duodenum, where it accounts for ~15% (Wang et al. 2017), of all reads or under disease states where its abundance may be enriched (Dubourg et al. 2013). Furthermore, *lactobacilli* largely localize in regions of the proximal intestine due to the ability to form biofilm‐like structures that enhance adherence (Walter 2008). Therefore, we postulate that a primary mechanism of action of UCC118 is the targeting of *Verrucomicrobia* and related subspecies in the gastroduodenal tract, which enhances protection against hyperpermeability. Supporting this notion, mice with a mutation leading to spontaneous irritable bowel syndrome display increased *Verrucomicrobiaceae* abundance (Schaubeck et al. 2016). Recently, Li et al. discovered that treatment with 5‐Fu, a potent inducer of colonic mucositis, also increases both fecal and cecum abundance of *Verrucomicrobia* (Li et al. 2017). However, healthy Chilean’s exhibit increased microbial abundance of *Verrucomicrobia* compared to obese age‐matched counterparts (Fujio‐Vejar et al. 2017), suggesting that the targetability of *Verrucomicrobia* in GI‐related disorders may be population and environment specific.

In addition to *Verrucomicrobia*, several other bacterial species relevant to GI health were differentially regulated. Both *Roseburia* and *Lachnospiraceae*, well characterized butyrate producing bacteria, were dramatically upregulated after treatment with UCC118. Short‐chain fatty acids, acetate, propionate and butyrate, are the primary fermentation byproducts of the gut microbiome. Butyrate is of high biological importance as it serves as the primary fuel source for the colonocyte and assists in modulating intestinal immune function. Butyrate also helps to maintain intestinal epithelial cell integrity, preventing disassociation of the tight junction proteins which seal the paracellular space and protect against macromolecule translocation (Ahmad et al. 2000; Smith et al. 2013). Furthermore, one of the most consistent findings among microbiome studies in patients with GI disease is alterations in levels of butyrate producing bacteria (Kostic et al. 2014). Multiple strains of bacteria positively associated with cardiovascular disease, such as *Streptococcus mutans* (*S. mutans*) and *Succinivibrio *, were reduced after supplementation (Nakano et al. 2006; Serena et al. 2018). Though the patients sequenced in this investigation were very healthy and free of confounding disorders, these findings suggest broader therapeutic application of UCC118.

Biomarkers of inflammation and tight junction activity remained unchanged following UCC118 supplementation. Previous research has shown reductions in zonulin and pro‐inflammatory cytokines after probiotic supplementation in humans (Lamprecht et al. 2012). IL‐10 knockout mice supplemented with UCC118 exhibited reduced GI inflammation (O'Mahony et al. 2001; Feighery et al. 2008). Additionally, infants with ectopic eczema supplemented with probiotic displayed significant reductions in severity of allergic inflammation (Isolauri et al. 2000). However, multiple well‐controlled clinical trials have failed to detect changes in inflammatory markers, despite improvements in primary therapeutic outcomes (Kajander et al. 2008; Worthley et al. 2009; Andreasen et al. 2010). Therefore, it may be that probiotic‐related clinical improvements in inflammatory disease are reflected in cellular, bacterial, and tissue remodeling, and to a lesser extent in the peripheral circulation.

Our results provide proof of principle that short‐term supplementation with UCC118 protects against exercise‐induced GI permeability in healthy humans. Furthermore, the favorable effects of UCC118 appear to rely on alterations in proximal small intestine permeability and microbiota. Further research would benefit from conducting large cohort safety and efficacy trials in clinical populations with extended supplementation to determine durability.

## Supporting information




**Table S1**: Differentially regulated bacteria after 4 weeks of supplementation with UCC118 by order and taxonomic group.
