# Supplementary material for: UCC118 supplementation reduces exercise‐induced gastrointestinal permeability and remodels the gut microbiome in healthy humans
Source: Physiol Rep. 2019 Nov 23;7(22):e14276. doi: 10.14814/phy2.14276 (PMC6874782; doi:10.14814/phy2.14276)
Supplement: Supplementary file 1 — Table S1: Differentially regulated bacteria after 4 weeks of supplementation with UCC118 by order and taxonomic group. [file PHY2-7-e14276-s001.docx]

Supplementary Table 1 – Differentially regulated bacteria after 4 weeks of supplementation with UCC118 by order and taxonomic group.

| **Order** | **Taxonomy** | **Abundance Pre UCC118** | **Abundance Post UCC118** | **Fold (log2)** | **p** |
| --- | --- | --- | --- | --- | --- |
| Class | d__Bacteria;k__Bacteria;p__Verrucomicrobia;c__Verrucomicrobiae | 3647.398 | 68.985 | -5.724 | 0.002 |
| Class | d__Bacteria;k__Bacteria;p__Proteobacteria;c__Epsilonproteobacteria | 251.261 | 2711.056 | 3.432 | 0.027 |
| Class | d__Eukaryota;k__Fungi;p__Basidiomycota;c__Pucciniomycetes | 5.559 | 30.573 | 2.459 | 0.028 |
| Class | d__Bacteria;k__Bacteria;p__Acidobacteria;c__Holophagae | 0.446 | 4.162 | 3.222 | 0.041 |
| Family | d__Bacteria;k__Bacteria;p__Verrucomicrobia;c__Verrucomicrobiae;o__Verrucomicrobiales;f__Verrucomicrobiaceae | 3652.176 | 58.124 | -5.973 | 0.001 |
| Family | d__Bacteria;k__Bacteria;p__Proteobacteria;c__Gammaproteobacteria;o__Legionellales;f__Legionellaceae | 0.000 | 8.320 | inf | 0.002 |
| Family | d__Bacteria;k__Bacteria;p__Proteobacteria;c__Gammaproteobacteria;o__Aeromonadales;f__Succinivibrionaceae | 454.343 | 15.009 | -4.920 | 0.004 |
| Family | d__Eukaryota;k__Fungi;p__Basidiomycota;c__Pucciniomycetes;o__Pucciniales;f__Pucciniaceae | 3.189 | 29.489 | 3.209 | 0.007 |
| Family | d__Viruses;k__Viruses;p__undef;c__undef;o__Caudovirales;f__Podoviridae | 4193.545 | 434.777 | -3.270 | 0.010 |
| Family | d__Bacteria;k__Bacteria;p__Proteobacteria;c__Epsilonproteobacteria;o__Campylobacterales;f__Campylobacteraceae | 252.219 | 3259.867 | 3.692 | 0.025 |
| Family | d__Eukaryota;k__Fungi;p__Basidiomycota;c__Agaricomycetes;o__Cantharellales;f__Tulasnellaceae | 4.981 | 23.884 | 2.262 | 0.028 |
| Family | d__Bacteria;k__Bacteria;p__Proteobacteria;c__Alphaproteobacteria;o__Sphingomonadales;f__Sphingomonadaceae | 54.096 | 10.005 | -2.435 | 0.031 |
| Genus | d__Eukaryota;k__Protozoa;p__undef;c__undef;o__Stemonitida;f__Stemonitidae;g__Diachea | 14.548 | 0.000 | -inf | 0.000 |
| Genus | d__Bacteria;k__Bacteria;p__Proteobacteria;c__Gammaproteobacteria;o__Aeromonadales;f__Succinivibrionaceae;g__Succinivibrio | 260.759 | 1.108 | -7.879 | 0.001 |
| Genus | d__Bacteria;k__Bacteria;p__Proteobacteria;c__Alphaproteobacteria;o__Rhodospirillales;f__Acetobacteraceae;g__Acidiphilium | 0.472 | 20.836 | 5.464 | 0.002 |
| Genus | d__Eukaryota;k__Protozoa;p__undef;c__undef;o__Stemonitida;f__Stemonitidae;g__Stemonitopsis | 0.000 | 6.667 | inf | 0.002 |
| Genus | d__Viruses;k__Viruses;p__undef;c__undef;o__Caudovirales;f__Myoviridae;g__P1virus | 25.664 | 703.506 | 4.777 | 0.002 |
| Genus | d__Bacteria;k__Bacteria;p__Proteobacteria;c__Gammaproteobacteria;o__Legionellales;f__Legionellaceae;g__Legionella | 0.000 | 9.166 | inf | 0.003 |
| Genus | d__Bacteria;k__Bacteria;p__Verrucomicrobia;c__Verrucomicrobiae;o__Verrucomicrobiales;f__Verrucomicrobiaceae;g__Prosthecobacter | 2936.757 | 60.952 | -5.590 | 0.004 |
| Genus | d__Viruses;k__Viruses;p__undef;c__undef;o__Caudovirales;f__Podoviridae;g__P22virus | 3944.995 | 200.582 | -4.298 | 0.004 |
| Genus | d__Bacteria;k__Bacteria;p__Firmicutes;c__Clostridia;o__Clostridiales;f__Lachnospiraceae;g__Catonella | 0.205 | 5.455 | 4.735 | 0.007 |
| Genus | d__Viruses;k__Viruses;p__undef;c__undef;o__Caudovirales;f__Podoviridae;g__Epsilon15virus | 7.007 | 184.480 | 4.719 | 0.022 |
| Genus | d__Viruses;k__Viruses;p__undef;c__undef;o__Caudovirales;f__Siphoviridae;g__C2virus | 3.279 | 54.757 | 4.062 | 0.022 |
| Genus | d__Eukaryota;k__Fungi;p__Basidiomycota;c__Pucciniomycetes;o__Pucciniales;f__Pucciniaceae;g__Puccinia | 3.106 | 29.349 | 3.240 | 0.023 |
| Genus | d__Bacteria;k__Bacteria;p__Proteobacteria;c__Gammaproteobacteria;o__Pseudomonadales;f__Moraxellaceae;g__Acinetobacter | 6.739 | 0.170 | -5.305 | 0.025 |
| Genus | d__Bacteria;k__Bacteria;p__Firmicutes;c__Bacilli;o__Bacillales;f__Bacillaceae;g__Psychrobacillus | 0.000 | 3.078 | inf | 0.028 |
| Genus | d__Bacteria;k__Bacteria;p__Proteobacteria;c__Epsilonproteobacteria;o__Campylobacterales;f__Campylobacteraceae;g__Campylobacter | 255.697 | 3575.279 | 3.806 | 0.033 |
| Genus | d__Viruses;k__Viruses;p__undef;c__undef;o__Caudovirales;f__Myoviridae;g__undef | 2713.510 | 505.439 | -2.425 | 0.041 |
| Genus | d__Bacteria;k__Bacteria;p__Proteobacteria;c__Gammaproteobacteria;o__Aeromonadales;f__Succinivibrionaceae;g__Ruminobacter | 58.933 | 2.401 | -4.618 | 0.043 |
| Genus | d__Bacteria;k__Bacteria;p__Proteobacteria;c__Gammaproteobacteria;o__Vibrionales;f__Vibrionaceae;g__Vibrio | 58.302 | 4.630 | -3.655 | 0.044 |
| Genus | d__Bacteria;k__Bacteria;p__Proteobacteria;c__Gammaproteobacteria;o__Enterobacterales;f__Enterobacteriaceae;g__Pluralibacter | 3.823 | 39.359 | 3.364 | 0.048 |
| Order | d__Bacteria;k__Bacteria;p__Verrucomicrobia;c__Verrucomicrobiae;o__Verrucomicrobiales | 3132.770 | 66.550 | -5.557 | 0.004 |
| Order | d__Bacteria;k__Bacteria;p__Proteobacteria;c__Gammaproteobacteria;o__Aeromonadales | 430.426 | 19.172 | -4.489 | 0.007 |
| Order | d__Eukaryota;k__Fungi;p__Basidiomycota;c__Pucciniomycetes;o__Pucciniales | 5.673 | 33.738 | 2.572 | 0.017 |
| Order | d__Bacteria;k__Bacteria;p__Proteobacteria;c__Alphaproteobacteria;o__Sphingomonadales | 51.502 | 9.873 | -2.383 | 0.019 |
| Order | d__Eukaryota;k__Fungi;p__Basidiomycota;c__Agaricomycetes;o__Cantharellales | 5.145 | 25.515 | 2.310 | 0.020 |
| Order | d__Bacteria;k__Bacteria;p__Proteobacteria;c__Epsilonproteobacteria;o__Campylobacterales | 266.219 | 3238.387 | 3.605 | 0.039 |
| Order | d__Eukaryota;k__Protozoa;p__undef;c__undef;o__Physariida | 0.000 | 1.602 | inf | 0.044 |
| Phylum | d__Bacteria;k__Bacteria;p__Verrucomicrobia | 2946.027 | 70.099 | -5.393 | 0.001 |
| Species | d__Bacteria;k__Bacteria;p__Actinobacteria;c__Actinobacteria;o__Corynebacteriales;f__Corynebacteriaceae;g__Corynebacterium;s__Corynebacterium_renale | 0.000 | 267.848 | inf | 0.000 |
| Species | d__Bacteria;k__Bacteria;p__Proteobacteria;c__Epsilonproteobacteria;o__Campylobacterales;f__Campylobacteraceae;g__Campylobacter;s__Campylobacter_jejuni | 2.585 | 2785.414 | 10.074 | 0.000 |
| Species | d__Viruses;k__Viruses;p__undef;c__undef;o__Caudovirales;f__Podoviridae;g__Epsilon15virus;s__Escherichia_phage_phiV10 | 0.000 | 156.399 | inf | 0.000 |
| Species | d__Bacteria;k__Bacteria;p__Firmicutes;c__Clostridia;o__Clostridiales;f__Lachnospiraceae;g__Roseburia;s__Roseburia_hominis | 0.305 | 272.399 | 9.804 | 0.001 |
| Species | d__Bacteria;k__Bacteria;p__Firmicutes;c__Bacilli;o__Lactobacillales;f__Streptococcaceae;g__Streptococcus;s__Streptococcus_mutans | 8876.772 | 24.612 | -8.495 | 0.001 |
| Species | d__Viruses;k__Viruses;p__undef;c__undef;o__Caudovirales;f__Siphoviridae;g__Lambdavirus;s__Escherichia_phage_HK639 | 0.000 | 46.891 | inf | 0.001 |
| Species | d__Viruses;k__Viruses;p__undef;c__undef;o__Caudovirales;f__Myoviridae;g__undef;s__Salmonella_phage_STML-13-1 | 0.000 | 38.907 | inf | 0.002 |
| Species | d__Viruses;k__Viruses;p__undef;c__undef;o__Caudovirales;f__Siphoviridae;g__undef;s__Salmonella_phage_vB_SosS_Oslo | 37.551 | 0.193 | -7.602 | 0.002 |
| Species | d__Viruses;k__Viruses;p__undef;c__undef;o__Caudovirales;f__Siphoviridae;g__Sfi11virus;s__Streptococcus_phage_858 | 19.949 | 0.000 | -inf | 0.002 |
| Species | d__Bacteria;k__Bacteria;p__Firmicutes;c__Bacilli;o__Lactobacillales;f__Enterococcaceae;g__Enterococcus;s__Enterococcus_malodoratus | 49.865 | 0.000 | -inf | 0.003 |
| Species | d__Eukaryota;k__Protozoa;p__undef;c__undef;o__Stemonitida;f__Stemonitidae;g__Diachea;s__Diachea_leucopodia | 14.740 | 0.000 | -inf | 0.003 |
| Species | d__Viruses;k__Viruses;p__undef;c__undef;o__Caudovirales;f__Podoviridae;g__P22virus;s__Enterobacteria_phage_CUS-3 | 3891.118 | 149.758 | -4.699 | 0.006 |
| Species | d__Bacteria;k__Bacteria;p__Firmicutes;c__Negativicutes;o__Veillonellales;f__Veillonellaceae;g__Veillonella;s__Veillonella_atypica | 0.382 | 42.122 | 6.786 | 0.006 |
| Species | d__Bacteria;k__Bacteria;p__Verrucomicrobia;c__Verrucomicrobiae;o__Verrucomicrobiales;f__Verrucomicrobiaceae;g__Prosthecobacter;s__Prosthecobacter_vanneervenii | 1026.338 | 10.455 | -6.617 | 0.006 |
| Species | d__Viruses;k__Viruses;p__undef;c__undef;o__Caudovirales;f__Siphoviridae;g__Sk1virus;s__Lactococcus_phage_CB13 | 0.000 | 9.430 | inf | 0.006 |
| Species | d__Bacteria;k__Bacteria;p__Proteobacteria;c__Gammaproteobacteria;o__Aeromonadales;f__Succinivibrionaceae;g__Succinivibrio;s__Succinivibrio_dextrinosolvens | 242.969 | 1.114 | -7.769 | 0.007 |
| Species | d__Eukaryota;k__Protozoa;p__undef;c__undef;o__Stemonitida;f__Stemonitidae;g__Stemonitopsis;s__Stemonitopsis_typhina | 0.000 | 6.968 | inf | 0.009 |
| Species | d__Viruses;k__Viruses;p__undef;c__undef;o__Caudovirales;f__Myoviridae;g__undef;s__Enterobacteria_phage_phiP27 | 21.034 | 0.735 | -4.839 | 0.010 |
| Species | d__Bacteria;k__Bacteria;p__Proteobacteria;c__Gammaproteobacteria;o__Vibrionales;f__Vibrionaceae;g__Vibrio;s__Vibrio_mediterranei | 47.716 | 0.580 | -6.362 | 0.010 |
| Species | d__Viruses;k__Viruses;p__undef;c__undef;o__Caudovirales;f__Siphoviridae;g__Lambdavirus;s__Enterobacteria_phage_cdtI | 530.004 | 18.633 | -4.830 | 0.010 |
| Species | d__Bacteria;k__Bacteria;p__undef;c__undef;o__undef;f__undef;g__undef;s__uncultured_eubacterium_E1-K15 | 6.083 | 0.000 | -inf | 0.011 |
| Species | d__Viruses;k__Viruses;p__undef;c__undef;o__Caudovirales;f__Myoviridae;g__P2virus;s__Yersinia_phage_L-413C | 91.483 | 3.307 | -4.790 | 0.013 |
| Species | d__Bacteria;k__Bacteria;p__Proteobacteria;c__Gammaproteobacteria;o__Legionellales;f__Legionellaceae;g__Legionella;s__Legionella_pneumophila | 0.000 | 7.926 | inf | 0.013 |
| Species | d__Viruses;k__Viruses;p__undef;c__undef;o__Caudovirales;f__Myoviridae;g__P1virus;s__Escherichia_virus_P1 | 28.015 | 707.274 | 4.658 | 0.014 |
| Species | d__Bacteria;k__Bacteria;p__Proteobacteria;c__Gammaproteobacteria;o__Enterobacterales;f__Yersiniaceae;g__Serratia;s__Serratia_symbiotica | 12.406 | 0.193 | -6.004 | 0.016 |
| Species | d__Bacteria;k__Bacteria;p__Actinobacteria;c__Actinobacteria;o__Corynebacteriales;f__Mycobacteriaceae;g__Mycobacterium;s__Mycobacterium_gastri | 4.633 | 0.000 | -inf | 0.017 |
| Species | d__Bacteria;k__Bacteria;p__Verrucomicrobia;c__Verrucomicrobiae;o__Verrucomicrobiales;f__Verrucomicrobiaceae;g__Prosthecobacter;s__Prosthecobacter_fusiformis | 1737.476 | 50.415 | -5.107 | 0.018 |
| Species | d__Viruses;k__Viruses;p__undef;c__undef;o__Caudovirales;f__Siphoviridae;g__Lambdavirus;s__Enterobacteria_phage_BP-4795 | 0.000 | 4.285 | inf | 0.018 |
| Species | d__Bacteria;k__Bacteria;p__Firmicutes;c__Bacilli;o__Lactobacillales;f__Enterococcaceae;g__Enterococcus;s__Enterococcus_avium | 73.177 | 1.252 | -5.869 | 0.018 |
| Species | d__Viruses;k__Viruses;p__undef;c__undef;o__Caudovirales;f__Podoviridae;g__P22virus;s__Salmonella_phage_HK620 | 0.649 | 26.309 | 5.342 | 0.019 |
| Species | d__Bacteria;k__Bacteria;p__Firmicutes;c__Clostridia;o__Clostridiales;f__Lachnospiraceae;g__Lachnoclostridium;s__[Clostridium]_indolis | 6.679 | 0.135 | -5.632 | 0.019 |
| Species | d__Bacteria;k__Bacteria;p__Proteobacteria;c__Gammaproteobacteria;o__Aeromonadales;f__Succinivibrionaceae;g__Anaerobiospirillum;s__Anaerobiospirillum_thomasii | 32.256 | 0.343 | -6.556 | 0.019 |
| Species | d__Bacteria;k__Bacteria;p__Proteobacteria;c__Alphaproteobacteria;o__Rhodospirillales;f__Acetobacteraceae;g__Acidiphilium;s__Acidiphilium_multivorum | 0.533 | 18.537 | 5.119 | 0.019 |
| Species | d__Viruses;k__Viruses;p__undef;c__undef;o__Caudovirales;f__Siphoviridae;g__Lambdavirus;s__Escherichia_virus_HK022 | 0.185 | 16.848 | 6.506 | 0.020 |
| Species | d__Bacteria;k__Bacteria;p__Actinobacteria;c__Actinobacteria;o__Streptomycetales;f__Streptomycetaceae;g__Streptomyces;s__Streptomyces_nodosus | 6.960 | 0.148 | -5.557 | 0.021 |
| Species | d__Viruses;k__Viruses;p__undef;c__undef;o__Caudovirales;f__Myoviridae;g__undef;s__Enterobacteria_phage_SfI | 892.236 | 53.290 | -4.065 | 0.022 |
| Species | d__Bacteria;k__Bacteria;p__Firmicutes;c__Clostridia;o__Clostridiales;f__Lachnospiraceae;g__Catonella;s__Catonella_sp._oral_clone_AH153 | 0.191 | 5.485 | 4.845 | 0.022 |
| Species | d__Bacteria;k__Bacteria;p__Proteobacteria;c__Gammaproteobacteria;o__Pasteurellales;f__Pasteurellaceae;g__Haemophilus;s__Haemophilus_pittmaniae | 14.933 | 0.193 | -6.271 | 0.023 |
| Species | d__Viruses;k__Viruses;p__undef;c__undef;o__Caudovirales;f__Siphoviridae;g__Lambdavirus;s__Enterobacteria_phage_mEp460 | 1483.773 | 94.827 | -3.968 | 0.026 |
| Species | d__Viruses;k__Viruses;p__undef;c__undef;o__Caudovirales;f__Siphoviridae;g__undef;s__Lactococcus_phage_jm2 | 5.969 | 0.135 | -5.470 | 0.030 |
| Species | d__Bacteria;k__Bacteria;p__Proteobacteria;c__Gammaproteobacteria;o__Pseudomonadales;f__Pseudomonadaceae;g__Pseudomonas;s__Pseudomonas_sp._KNA6-5 | 0.000 | 5.220 | inf | 0.030 |
| Species | d__Viruses;k__Viruses;p__undef;c__undef;o__undef;f__undef;g__undef;s__Streptococcus_phage_phi1207.3 | 0.388 | 7.859 | 4.338 | 0.031 |
| Species | d__Bacteria;k__Bacteria;p__Proteobacteria;c__Alphaproteobacteria;o__Rhizobiales;f__Bartonellaceae;g__Bartonella;s__Bartonella_quintana | 30.920 | 0.887 | -5.124 | 0.031 |
| Species | d__Bacteria;k__Bacteria;p__Firmicutes;c__Negativicutes;o__Selenomonadales;f__Selenomonadaceae;g__Selenomonas;s__Selenomonas-like_sp._oral_strain_FNA3 | 3.269 | 0.000 | -inf | 0.032 |
| Species | d__Viruses;k__Viruses;p__undef;c__undef;o__Caudovirales;f__Myoviridae;g__undef;s__Shigella_phage_SfIV | 1240.020 | 102.575 | -3.596 | 0.033 |
| Species | d__Bacteria;k__Bacteria;p__Proteobacteria;c__Gammaproteobacteria;o__Pseudomonadales;f__Moraxellaceae;g__Acinetobacter;s__Acinetobacter_baumannii | 4.390 | 0.000 | -inf | 0.039 |
| Species | d__Viruses;k__Viruses;p__undef;c__undef;o__Caudovirales;f__Siphoviridae;g__C2virus;s__Lactococcus_phage_bIL67 | 1.471 | 35.837 | 4.607 | 0.039 |
| Species | d__Eukaryota;k__Fungi;p__Basidiomycota;c__Pucciniomycetes;o__Pucciniales;f__Pucciniaceae;g__Puccinia;s__Puccinia_striiformis | 1.652 | 26.860 | 4.023 | 0.040 |
| Species | d__Eukaryota;k__Fungi;p__Ascomycota;c__Saccharomycetes;o__Saccharomycetales;f__Saccharomycetaceae;g__Saccharomyces;s__Saccharomyces_bayanus | 0.726 | 11.961 | 4.042 | 0.041 |
| Species | d__Bacteria;k__Bacteria;p__Firmicutes;c__Clostridia;o__Clostridiales;f__Lachnospiraceae;g__Blautia;s__[Ruminococcus]_torques | 0.556 | 11.386 | 4.356 | 0.043 |
| Species | d__Viruses;k__Viruses;p__undef;c__undef;o__Caudovirales;f__Siphoviridae;g__Lambdavirus;s__Enterobacteria_phage_mEpX1 | 0.000 | 4.255 | inf | 0.043 |
| Species | d__Bacteria;k__Bacteria;p__Proteobacteria;c__Alphaproteobacteria;o__Sphingomonadales;f__Sphingomonadaceae;g__Sphingomonas;s__Sphingomonas_sp._KT0216 | 44.836 | 2.998 | -3.902 | 0.043 |
| Species | d__Bacteria;k__Bacteria;p__Firmicutes;c__Clostridia;o__Clostridiales;f__Lachnospiraceae;g__Tyzzerella;s__[Clostridium]_neopropionicum | 3.630 | 0.000 | -inf | 0.044 |
| Species | d__Bacteria;k__Bacteria;p__Firmicutes;c__Clostridia;o__Clostridiales;f__undef;g__undef;s__Elbe_River_snow_isolate_Iso15_5 | 3.572 | 0.000 | -inf | 0.045 |
| Species | d__Bacteria;k__Bacteria;p__Proteobacteria;c__Gammaproteobacteria;o__Enterobacterales;f__Enterobacteriaceae;g__Klebsiella;s__Klebsiella_sp. | 380.096 | 36.130 | -3.395 | 0.045 |
| Species | d__Bacteria;k__Bacteria;p__Actinobacteria;c__Actinobacteria;o__Micrococcales;f__Intrasporangiaceae;g__Tetrasphaera;s__Tetrasphaera_japonica | 0.558 | 8.518 | 3.933 | 0.046 |
| Species | d__Viruses;k__Viruses;p__undef;c__undef;o__Caudovirales;f__Siphoviridae;g__Lambdavirus;s__Escherichia_virus_HK97 | 2.594 | 0.000 | -inf | 0.046 |
| Species | d__Viruses;k__Viruses;p__undef;c__undef;o__Caudovirales;f__Myoviridae;g__Felixo1virus;s__Salmonella_phage_FelixO1 | 8.339 | 1.102 | -2.919 | 0.047 |
| Species | d__Bacteria;k__Bacteria;p__Firmicutes;c__Bacilli;o__Lactobacillales;f__Lactobacillaceae;g__Lactobacillus;s__Lactobacillus_acidophilus | 1.488 | 29.539 | 4.311 | 0.049 |
| Species | d__Viruses;k__Viruses;p__undef;c__undef;o__Caudovirales;f__Siphoviridae;g__Lambdavirus;s__Enterobacteria_phage_HK633 | 2.502 | 0.000 | -inf | 0.049 |
